# Adenoma detection rate using narrow-band imaging is inferior to high-definition white light colonoscopy in screening and surveillance colonoscopies in daily clinical care: A randomized controlled trial

**DOI:** 10.1097/MD.0000000000029858

**Published:** 2022-08-12

**Authors:** Martin Bürger, Marko Weber, Iver Petersen, Andreas Stallmach, Carsten Schmidt

**Affiliations:** a Department of Gastroenterology, Hepatology and Infectious Diseases, Clinic for Internal Medicine IV, Jena University Hospital, Jena, Germany; b Clinic for Gastroenterology and Hepatology, Faculty of Medicine and University Hospital Cologne, University of Cologne, Cologne, Germany; c Institute of Pathology, Jena University Hospital, Jena, Germany; d Institute of Pathology, Waldklinikum Gera, Gera, Germany; e Department of Gastroenterology, Hepatology, Endocrinology, Diabetes and Infectious Diseases, Medical Clinic II, Fulda Hospital, Fulda, Germany; f Medical Faculty of the Friedrich Schiller University, Jena, Germany.

**Keywords:** adenoma detection rate, colonoscopy, colorectal cancer, narrow-band imaging, polyps

## Abstract

**Background:**

Despite recent advances in endoscopic technology adenoma miss rate still is up to 20% contributing to interval cancers. Improved imaging modalities have been introduced to increase adenoma detection rate (ADR). Recently, narrow-band imaging (NBI) (Exera II series, Olympus Corporation) was not significantly better than high-definition white light colonoscopy (HD-WLC). An improved second generation of NBI (190-NBI) is characterized by better illumination of the bowel lumen and may be associated with a higher ADR.

**Methods:**

We performed a prospective randomized study on patients referred to the Jena University Hospital for screening or surveillance colonoscopy between January 2015 and April 2017. Participating endoscopists were divided into 2 subgroups depending on their individual experience. Colonoscopy was performed by use of HD-WLC or 190-NBI upon withdrawal.

**Results:**

Five hundred fifty-three patients participated in the study. Eighty patients were excluded (insufficient bowel cleansing [n = 34], anticoagulation precluding polypectomy [n=15], partial colonic resection [n=9], other reasons [n = 22]). Mean age was 66.9 years (standard deviation 10.3 years), and 253 patients were male (53.5%). Bowel preparation and withdrawal time were not different. ADR among all subgroups was 39.4% using HD-WLC, but only 29.1% were using 190-NBI (*P* = .02). Number of polyps per patient was lower using 190-NBI than with HD-WLC (0.58 vs 0.86; *P* = .02). Subgroup analysis revealed that 190-NBI was inferior to HD-WLC only in unexperienced endoscopists.

**Conclusion:**

In our stud,y ADR was lower by use of 190-NBI. These differences persisted only in unexperienced investigators. 190-NBI seems to be more challenging regarding ADR, requiring more intensive training prior to implementing this technology in daily clinical care.

**Registration:**

ClinicalTrials.gov (identifier: NCT03081975).

## 1. Introduction

Colorectal cancer (CRC) is the world’s third most frequent cancer and second leading cause of cancer-associated mortality worldwide.^[1]^ Screening and subsequent surveillance colonoscopies are efficient tools to prevent CRC. Because most CRCs arise through preexisting adenomas^[2]^ either through adenoma-carcinoma sequence or the serrated pathway,^[3]^ screening colonoscopies significantly reduce the incidence of bowel cancer in individuals aged >55 years.^[4]^

Despite recent advances in endoscopic technology, adenoma miss rate still is up to 20% and contributes to the occurrence of interval cancers.^[5]^ Therefore, improved imaging modalities have been introduced to increase adenoma detection rate (ADR) during screening and surveillance colonoscopies. Especially sessile serrated adenomas are considered having high potential to transform into CRC^[6,7]^ and contribute to interval cancers^[8]^ because of their special morphology coming along with a more difficult detection during colonoscopy.^[9]^ As recommendations of surveillance intervals are based on characteristics of adenomas found on screening or surveillance colonoscopies,^[10]^ an optimal ADR is crucial for the patient.

In recent years, different methods and techniques were introduced to possibly enhance adenoma and PDR, from chromoendoscopy, cap-assisted colonoscopy to improve mucosal visualization to different image-enhancing modules such as “Fuji Intelligent Color Enhancement” (Fujinon Inc., Saitama, Japan), “Narrow-band imaging” (190-NBI) (Olympus Inc., Tokyo, Japan), “iSCAN” (HOYA Corporation, PENTAX Lifecare, Tokyo, Japan), for example,^[11]^ 190-NBI is a widely available optical filter modality that has been incorporated into Exera III series endoscopes that is thought to help identify colorectal neoplasms in vivo.^[12]^

NBI is a virtual chromoendoscopy modality using an optical filter focusing on blue (415 nm) and green (540 nm) light, thus targeting the highest absorption peak of hemoglobin, resulting in enhanced visualization of blood vessels and mucosal surface patterns. As shown by several studies, 190-NBI is a useful tool to further characterize colonic polyps, to discriminate neoplastic from nonneoplastic lesions,^[12]^ and to endoscopically predict deep submucosal invasive carcinoma.^[13]^ It might have the potential to replace pathological diagnoses of diminutive polyps^[14]^ and to improve identification of dysplasia or cancer in patients with Barrett´s esophagus.^[15]^ Recently, 190-NBI was already implemented in international guidelines for neoplasia characterization by the European Society of Gastrointestinal Endoscopy (ESGE)^[16]^ and the American Gastroenterological Association.^[17]^

However, first-generation NBI was not significantly better than high-definition white light colonoscopy (HD-WLC) for the detection of patients with colorectal polyps or colorectal adenomas.^[18,19]^ But, an improved second generation of 190-NBI is characterized by better illumination of the bowel lumen in combination with high-definition imaging. Consequently, 190-190-NBI may be associated with a higher ADR than high-definition HD-WLC alone in average-risk individuals.^[11]^ Therefore, there is a need for a prospective randomized study with regard to the performance of 190-NBI concerning adenoma detection in daily clinical practice, particularly with the involvement of doctors in training.

In this randomized controlled study at a tertiary care hospital, we prospectively compared adenoma and PDR using either HD white light endoscopy or 190-NBI-190 chromoendoscopy in consecutive screening and surveillance colonoscopies. In particular, we evaluated the performance of unexperienced endoscopists in using HD-WLC vs 190-NBI chromoendoscopy.

## 2. Methods

### 2.1. Patients

In this prospective randomized controlled trial, a total of 553 consecutive patients referred for screening or surveillance colonoscopy were randomized after written informed consent was given. Screening colonoscopy was added to the German national statutory cancer screening program in October 2002^[4]^; therefore, patients were aged ≥55 for screening colonoscopies. For surveillance colonoscopies, patients had to be aged ≥18 years with a clinical need for surveillance. Exclusion criteria were patients aged <18 years, women with potential or existing pregnancy, and patients who declined to participate in the study.

### 2.2. Study design

This randomized controlled trial was performed at the Department of Interdisciplinary Endoscopy at Jena University Hospital to compare HD-WLC and 190-NBI in screening and surveillance colonoscopies upon withdrawal.

The endoscopic procedure was assigned on the basis of a computer-generated randomization list, so that 50% of the patients were examined by means of 190-NBI imaging and 50% of the patients by white light endoscopy. Patient assignment was performed by a study nurse who was not involved in the endoscopic procedures. Endoscopists were divided into an unexperienced and an experienced group, depending on the number of individually performed colonoscopies (<450 or ≥450 colonoscopies), as Munroe and co-workers^[20]^ have previously shown that this number of procedures is necessary to yield acceptable adenoma miss rates. Both groups were previously trained through an online tool to become familiar with 190-NBI imaging (http://www.nbi-training.eu/) and were familiarized with the technology by at least 10 individual examinations before the beginning of the study. Every participating endoscopist had performed a minimum of 100 self-conducted colonoscopies prior to participation in the study. Study period was the duration of the colonoscopy.

### 2.3. Intervention and techniques

Before colonoscopy, all patients were given dietary instructions. For bowel cleansing, a split-dose regimen with 4 L of polyethylene glycol was used, starting the day before the procedure. Conscious sedation was performed with intravenous midazolam and propofol on patient’s preference. All colonoscopies were performed with Olympus CF-HQ190 colonoscopes, the EVIS-EXERA III CLV-190 video system (Olympus Optical, Tokyo, Japan) and by using high-definition monitors. After reaching the cecum using HD-WLC, withdrawal was performed with either 190-NBI or HD-WLC. Withdrawal time started with switching to the randomized examination technique for the complete colonic visualization and included polypectomies. According to German guidelines, a minimum of 6 minutes was set for withdrawal. Bowel preparation was objectified by using the Boston Bowel Preparation Scale (BBPS).^[21]^ Because bowel preparation is critical for polyp detection rate (PDR) and ADR, we used a strict interpretation of the BBPS per colonic segment to assess bowel cleansing and created groups: “excellent” with a minimum of ≥2 in each bowel segment, “good” with a minimum of ≥1 and “insufficient” in case of a bowel segment assessed with 0. The latter group of patients was excluded from further analysis. If polypectomy was performed, all polyps were documented concerning location, size, and histological result.

### 2.4. Sample size determination

An ADR at white light endoscopy of 20% was assumed. Data using the new generation of 190-NBI technology were not yet available. In previous studies, an increase in ADR of 8% to 9% was assumed, resulting in a case number estimation (ADR of HD-WLC 20% vs 190-NBI 28.5% ADR, power 80%, α-error 0.05, 1-sided test) of 314 patients per group. An interim analysis revealed inferiority of 190-NBI vs HD-WLC without the statistical computational possibility for change, which led to the termination of the study.

### 2.5. Study endpoints

The primary endpoint was ADR using either HD-WLC or 190-NBI. Secondary endpoints were PDRs, number of adenomas and polyps per patient, and procedure time. Furthermore, we wanted to assess the influence of the endoscopists’ experience using 190-NBI concerning the ADR.

### 2.6. Statistical analysis

Categorical data were summarized as the percentage of the group total. Results were described as median and range or mean and standard deviation (SD), as appropriate. Associations of parametric continuous data were evaluated using the *t* test or the Wilcoxon rank-sum test (for nonparametric data). Fisher exact test (2-sided) was used to explore associations of categorical data between 2 groups. The Kruskal–Wallis test was used to assess group differences with >2 categories. A *P* value of <.05 was considered statistically significant. Results were calculated using the IBM SPSSwin^®^ Statistics software, version 24 (Somers, NY).

### 2.7. Ethical statement

The study was approved by the ethics committee of Jena University Hospital (No. 4302-01/15) and was performed in accordance with the ethical standards laid down in the 1964 Declaration of Helsinki and its later amendments. The study was registered at ClinicalTrials.gov (identifier: NCT03081975).

## 3. Results

### 3.1. Patient characteristics

**Figure 1.**
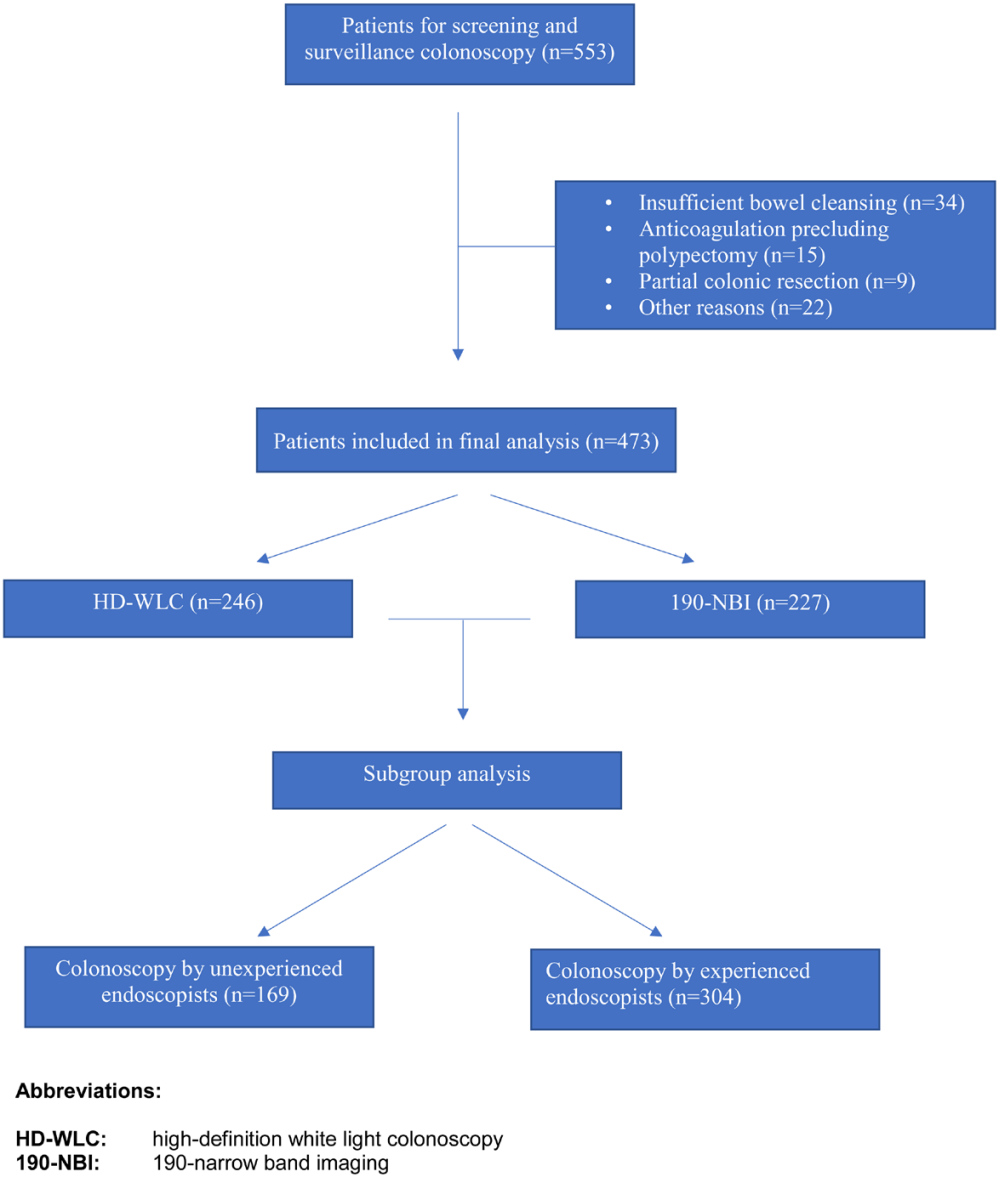
Flow sheet of screened patients. HD-WLC = high-definition white light colonoscopy, 190-NBI = 190-narrow-band imaging.

In the study period from March 2015 to June 2017, a total of 5644 colonoscopies were performed at the Department of Interdisciplinary Endoscopy at Jena University Hospital. A total of 553 patients were included in the study, and a total of 80 patients were excluded from further analysis (insufficient bowel cleansing (n = 34), anticoagulation precluding polypectomy (n=15), partial colonic resection (n = 9), other reasons (n = 22) including failed cecal intubation, and patients with familial CRC syndrome (hereditary nonpolyposis CRC syndrome). Therefore, 473 patients were included in the final analysis. In 402 patients, screening colonoscopy was performed, while 71 patients presented for surveillance after prior removal of adenomatous polyps. Two hundred forty-six patients were included in the HD-WLC group and 227 in the 190-NBI group, respectively. Mean age of patients was 66.9 years (SD 10.3 years), and 253 patients were male (53.5%). One hundred sixty-nine colonoscopies were performed by unexperienced investigators, while 304 colonoscopies were performed by experienced endoscopists, respectively (see Fig. 1 and Table 1).

**Table 1:** Patients and procedure characteristics.

	HD-WLC (n = 246)	190-NBI (n = 227)	*P* value
Mean age (SD), yr	67.4 (10.7)	66.4 (9.9)	.31
Sex, male, n (%)	124 (50.4)	129 (56.8)	.168
Indication for colonoscopy, n (%)			
Screening	216 (87.8)	186 (81.9)	.094
Surveillance	30 (12.2)	41 (18.1)	
Bowel preparation quality, n (%)			
Excellent	185 (75.2)	184 (81.1)	.149
Good	61 (24.8)	43 (18.9)	
Mean withdrawal time (SD), min	17.6 (12.6)	16.8 (9.6)	.108
Endoscopist, n (%)			
Experienced	159 (64.6)	145 (63.9)	.924
Nonexperienced	87 (35.4)	82 (36.1)	

### 3.2. Procedure characteristics

Cleansing score according to BBPS was ≥2 in each segment in 369 patients (78%), and in 104 patients (22%), a segment score of 1 was present with no significant difference between both groups. Mean withdrawal time was 17.2 minutes (SD 11.3 minutes), including polypectomies. There were no statistically significant differences in mean withdrawal between HD-WLC (17.6 minutes; SD 12.6 minutes) and 190-NBI (16.8 minutes, SD 9.6 minutes; *P* = .108; see also Table 1). No serious adverse events were reported among both groups during the entire study.

### 3.3. Polyp characterization

Most of the polyps were found in the left colon (55.4%), as were most of the adenomas (63.3%). In the right colon, 31.0% of the polyps and 29.3% of adenomas were detected, and 13.1% of polyps and only 0.07% of adenomas were removed from the rectum. With regard to the polyp size, no information was provided in 5% of the polyps, 58% were between 0 and 5 mm in size, 18% were 6 to 9 mm, and 19% were ≥10 mm (mean: 6.5 mm; SD: 5.5 mm; min: 1 mm; max: 50 mm). Morphological appearance of the polyps was mostly diminutive polyps (53%), in 7% pedunculated and in 38% sessile polyps (no information provided in 2%). Histological findings of removed polyps are summarized in Table 2.

**Table 2:** Histological reports on removed polyps.

Histology	n (%)	HD-WLC	190-NBI
Tubular adenoma	293 (46.0)	181 (61.8%)	112 (38.2%)
Hyperplastic polyp	154 (24.2)	62 (40.3%)	92 (59.7%)
Pseudo-polyps	101 (15.9)	55 (54.5%)	46 (45.5%)
Serrated adenoma	38 (6.0)	22 (57.9%)	16 (42.1%)
Tubulovillous adenoma	14 (2.2)	10 (71.4%)	4 (28.6%)
Carcinoma	3 (0.5)	1 (33.3%)	2 (66.7%)
Villous adenoma	0 (0)	0 (0%)	0 (0%)
Other	2 (0.3)	2 (100%)	0 (0%)

Six polyps (0.9%) were lost precluding histological evaluation, while 26 polyps (4.1%) were left in the colon due to a high number of polyps already removed. These polyps were to be removed in a subsequent colonoscopy, independent of the study.

### 3.4. Adenoma and PDRs

Adenoma detection rate (ADR) among all subgroups was 39.4% by use of HD-WLC, but only 29.1% using 190-NBI (*P* = 0.02). Moreover, number of adenomas per patient was lower by use of 190-NBI than with HD-WLC (0.59 vs 0.87; *P* = .02). PDR was not significantly different between groups (53.3% by use of HD-WLC vs 49.8% using 190-NBI (*P* = .463)).

Subgroup analysis revealed that 190-NBI was inferior to HD-WLC with regard to ADR only in unexperienced endoscopists (22.5% vs 45.3%, *P* = .003), while there were no such differences in experienced investigators (32.7% vs 36.3%; *P* = .549; see Table 3). Moreover, PDR was also reduced in unexperienced endoscopists using 190-NBI (38.8% vs 57%; *P* = .021), while there was no difference in experienced investigators (55.8% vs 51.3%; *P* = .492). Data are summarized in Table 3.

**Table 3:** Adenoma detection rate, polyp detection rate, number of adenomas per patient, and subgroup analysis with regard to investigators experience.

	All endoscopists (n = 473)				Experienced endoscopists (n = 304)	Nonexperienced endoscopists (n = 169)
	Adenoma detection rate	Polyp detection rate	Number of adenomas per patient		Adenoma detection rate	
HD-WLC	39.4%	53.3%	0.87	36.3%		45.3%
190-NBI	29.1%	49.8%	0.59	32.7%		22.5%
*P* value	.020	.463	.020	.549		.003

Unfortunately, adequate bowel cleansing was lower than expected. Since adequate bowel cleansing is crucial for PDR and ADR, we did a separate calculation of the patient groups regarding the bowel preparation with “excellent” and “good” bowel cleansing: Separate calculations are significantly in line with the overall calculation in terms of ADR regarding investigators experience. In contrast, although ADR does not show any significant differences for all endoscopists, it does show a clear numerical trend congruent to the combined calculation, even though not statistically significant. This of statistical significance can be explained by the missing power due to the by separating calculations resulting small number of patients (data shown in Tables S1 and S2, Supplemental Digital Content, http://links.lww.com/MD/G867).

## 4. Discussion

A substantial number of techniques have been developed in order to increase ADR.^[22,23]^ NBI is one of the most widely available imaging-enhancing modules, but it remains controversial if it helps to improve the rate of polyp detection and particularly adenoma detection. While several individual prior studies evaluating Olympus’ first-generation 190-NBI methodology (Exera II series) have not shown improvement regarding ADR compared to HD-WLC,^[18,19,24–26]^ a recent meta-analysis of data from individual patients in randomized controlled trials summed up an advantage in terms of ADR including 3 studies using the current Exera III series.^[27]^ One of the explanations for 190-NBI inferiority was a reduced illumination associated with 190-NBI imaging. The current Exera III series is equipped with a significantly improved illumination of the intestinal lumen. However, recent studies yielded conflicting results concerning ADR of 190-NBI methodology,^[26,28–33]^ while the meta-analysis of Atkinson and co-workers^[27]^ including 3 RCTs using second-generation bright 190-NBI revealed an enhanced ADR (odds ratio 1.28; *P* = .02), especially when colon preparation was best.

In our randomized prospective study, we compared the performance of 190-NBI with HD-WLC on colorectal ADR in screening and surveillance colonoscopies performed by experienced and unexperienced endoscopists at a tertiary center. The most important finding of our study is a significant difference in ADR which was 39.4% by use of HD-WLC, but only 29.1% using 190-NBI (*P* = .02). The difference of approximately 10% appears to be clinically relevant because ADR is inversely associated with the risks of colorectal interval cancer.^[34]^ Concerning PDR, use of 190-NBI (49.8%) was also numerically inferior HD-WLC (53.3%), and in line with these results, number of adenomas per patient was lower by use of 190-NBI than with HD-WLC (0.59 vs 0.87; *P* = .02).

In a subgroup analysis, this inferiority of 190-NBI persisted only in unexperienced investigators with an ADR of 22.5% vs 45.3% when using HD-WLC (*P* = .003). Thus, unexperienced endoscopists using 190-NBI did not meet the quality requirements of an ADR of 25% as stated in the ESGE guidelines.^[35]^ Interestingly, we observed no inferiority of 190-NBI compared to HD-WLC in experienced investigators (*P* = .549). In line, PDR was also reduced in unexperienced endoscopists using 190-NBI (*P* = .02), while it was comparable in experienced investigators (*P* = .492).

With regard to the aforementioned meta-analysis consisting 3 RCTs using 190-NBI, colonoscopies in the study by Rex and co-workers^[36]^ were performed only by experienced, board-certified gastroenterologists, not showing an increase in the detection of proximal colon serrated lesions (*P* = .085). Likewise, Horimatsu et al did not find an increase in ADR (low and high grade), while number of polyps increased when using 190-NBI (2.01 vs 1.56; *P* = .032). However, in a subgroup analysis of endoscopists (highly experienced or experienced in 190-NBI or trainees), no such difference has been found, presumably due to the small sample size.^[37]^ Interestingly, in the study by Leung and co-workers,^[32]^ ADR was increased with 190-NBI imaging compared to white light endoscopy in experienced endoscopists (53.9% vs 34.7%; *P* = .018), while there was no significant difference among fellows (42.9% vs 34.3%; *P* = .24).

In summary, we conclude that 190-NBI seems to be more challenging with regard to adenoma detection, especially for less experienced endoscopists, requiring more intensive training prior to implementing this technology into daily clinical care, even if the number of investigations required has not been evaluated yet. However, even in experienced investigators, 190-NBI did not further increase ADR as compared to high-definition white light imaging in our study.

There are several factors beyond the endoscopists’ experience and imaging modality that have been associated with ADR such as withdrawal time,^[38]^ quality of bowel preparation, sex, and age.^[39]^ While randomization led to equal distribution of sex and age among investigated patients, quality of bowel preparation was not significantly different between patient groups. Moreover, as colonoscopy withdrawal time correlates with higher colon PDRs,^[40]^ we set the minimum withdrawal time to 6 minutes, which was reached in every performed colonoscopy, also not being different between 190-NBI and HD-WLC group. Finally, we used the BBPS^[21]^ according to the ESGE Guidelines defining a bowel preparation as adequate when BBPS ≥6^[35]^ is documented, with no difference between both groups in terms of bowel preparation.

Our study has several strengths as well as limitations. A major strength is our large, prospectively randomized patient population that reflects a real-life setting within a western endoscopy unit run by both faculty and trainee fellows. Moreover, endoscopists yielded a remarkably high ADR using HD-WLC indicating high-quality performance of colonoscopy, and therefore facilitating a comparison with 190-NBI. Conversely, a major limitation is the participation of both experienced and unexperienced investigators, resulting in rather inconsistent results. Prior experience with 190-NBI in the latter group was low, limiting the validity of results using 190-NBI. Finally, even if not suitable for daily clinical care, a back-to-back study design with subsequent examinations with both HD-WLC and 190-NBI may have been advantageous to compare imaging modalities at the level of the individual patient.

In summary, our randomized controlled study showed that 190-NBI is inferior to HD-WLC regarding ADR in the entire study group, but especially in nonexperienced endoscopists. Even in experienced hands, 190-NBI did not offer advantages over HD-WLC, making this imaging modality dispensable in daily clinical care.

## Acknowledgments

An abstract of this manuscript has been presented as conference abstract with the title “Adenoma detection rate using narrow band imaging surprisingly is inferior to high-definition white light colonoscopy in screening and surveillance colonoscopies performed by unexperienced endoscopists” with the assistance of Verena Sauer and Jessica Rüddel.

## Author contributions

Martin Bürger:

-Conception and design

-Acquisition of data

-Analysis of data

-Endoscopic investigations

-Interpretation of data

-Drafting the work

-Final approval of the manuscript

Marko Weber:

-Acquisition of data

-Interpretation of data

-Revising the work

-Final approval of the manuscript

Andreas Stallmach:

-Acquisition of data

-Endoscopic investigations

-Interpretation of data

-Revising the work

-Final approval of the manuscript

Iver Petersen:

-Analysis of specimen

-Interpretation of data

-Revising the work

-Final approval of the manuscript

Carsten Schmidt:

-Conception and design

-Acquisition of data

-Endoscopic investigations

-Analysis of data

-Interpretation of data

-Drafting the work

-Final approval of the manuscript

-Statistical analysis

## Supplementary Material


