# Supplementary material for: Adenoma detection rate using narrow-band imaging is inferior to high-definition white light colonoscopy in screening and surveillance colonoscopies in daily clinical care: A randomized controlled trial
Source: Medicine (Baltimore). 2022 Aug 12;101(32):e29858. doi: 10.1097/MD.0000000000029858 (PMC9371537; doi:10.1097/MD.0000000000029858)
Supplement: Supplementary file 1 [file medi-101-e29858-s001.pdf]

Supplementary material

Title: Adenoma detection rate using narrow band imaging is inferior to high-definition white light colonoscopy in screening and surveillance colonoscopies in daily clinical care: a randomized controlled trial

Authors: Martin Bürger, Marko Weber, Iver Petersen, Andreas Stallmach, Carsten Schmidt

Content: supplementary table S1 and supplementary table S2

Supplementary table S1.: Adenoma detection rate, polyp detection rate, number of adenomas per patient and subgroup analysis with regard to investigators experience for “BBPS excellent” group.

BBPS excellent

	All endoscopists (n= 369)			Experienced endoscopists (n=237)	Non-experienced endoscopists (n=132)
	Adenoma detection rate	Polyp detection rate	Number of adenomas per patient	Adenoma detection rate	
**HD-WLC**	39.5%	54.6%	0.9	35.8%	46.2%
**190-NBI**	31,0%	53.3%	0.61	35.0%	23.9%
**P**	0.102	0.835	0.047	1	0.01

Abbreviations:

BBPS: Boston bowel preparation scale
HD-WLC: high-definition white light colonoscopy
190-NBI: 190-narrow band imaging

Supplementary table S2.: Adenoma detection rate, polyp detection rate, number of adenomas per patient and subgroup analysis with regard to investigators experience for “BBPS good” group.

BBPS good

	All endoscopists (n= 104)			Experienced endoscopists (n=70)	Non-experienced endoscopists (n=34)
	Adenoma detection rate	Polyp detection rate	Number of adenomas per patient	Adenoma detection rate	
**HD-WLC**	39.3%	49.2%	0.75	37.5%	42.9%
**190-NBI**	20.9%	34.9%	0.44	23.3%	15.4%
**P**	0.056	0.165	0.162	0.299	0.14

Abbreviations:

BBPS: Boston bowel preparation scale
HD-WLC: high-definition white light colonoscopy
190-NBI: 190-narrow band imaging

Supplementary material legend

Supplementary table S1.: Adenoma detection rate, polyp detection rate, number of adenomas per patient and subgroup analysis with regard to investigators experience for “BBPS excellent” group.

Supplementary table S2.: Adenoma detection rate, polyp detection rate, number of adenomas per patient and subgroup analysis with regard to investigators experience for “BBPS good” group.
